# Antisense oligonucleotide–mediated inhibition of angiopoietin-like protein 3 increases reverse cholesterol transport in mice

**DOI:** 10.1016/j.jlr.2021.100101

**Published:** 2021-08-06

**Authors:** Thomas A. Bell, Mingxia Liu, Aaron J. Donner, Richard G. Lee, Adam E. Mullick, Rosanne M. Crooke

**Affiliations:** Cardiovascular Antisense Drug Discovery Group, Ionis Pharmaceuticals, Inc., Carlsbad, CA, USA

**Keywords:** antisense oligonucleotide, ANGPTL3, atherosclerosis, cholesterol, coronary artery disease, HDL, lipids, reverse cholesterol transport, hyperlipidemia, cardiovascular disease, ^3^H-CHE, ^3^H-cholesteryl hexadecyl ether, ANGPTL3, angiopoietin-like protein 3, ASCVD, atherosclerotic cardiovascular disease, ASO, antisense oligonucleotide, EL, endothelial lipase, FCR, fractional catabolic rate, FHBL2, familial combined hypobetalipoproteinemia, LOF, loss-of-function, LSC, liquid scintillation counting, RCT, reverse cholesterol transport, tg, transgenic, TPC, total plasma cholesterol, WD, Western diet

## Abstract

Supported by an abundance of experimental and genetic evidence, angiopoietin-like protein 3 (ANGPTL3) has emerged as a promising therapeutic target for cardiovascular disease. ANGPTL3 is primarily produced by the liver and is a potent modulator of plasma lipids and lipoproteins. Experimental models and subjects with loss-of-function *Angptl3* mutations typically present with lower levels of HDL-C than noncarriers. The effect of ANGPTL3 on HDL-C is typically attributed to its function as an inhibitor of the enzyme endothelial lipase. The ability to facilitate reverse cholesterol transport (RCT), the transport of cholesterol from peripheral tissues back to the liver, is a proposed antiatherogenic property of HDL. However, the effect of ANGPTL3 inhibition on RCT remains unclear. Here, we performed a series of dose-response and RCT studies using an *Angptl3* antisense oligonucleotide (ASO) in mouse models with varying plasma lipid profiles ranging from moderately to severely hyperlipidemic. *Angptl3* ASO-mediated reduction in HDL-C was limited to the model with moderate lipidemia, where the majority of plasma cholesterol was associated with HDL. Surprisingly, regardless of the effect on HDL-C, treatment with the *Angptl3* ASO enhanced RCT in all models tested. The observations from the RCT assays were confirmed in HDL clearance studies, where mice treated with the *Angptl3* ASO displayed increased plasma clearance and hepatic uptake of labeled HDL. The results from our studies suggest that inhibition of ANGPTL3 not only reduces levels of proatherogenic lipids but also improves HDL-mediated RCT.

Despite advances in education, detection, and treatment, atherosclerotic cardiovascular disease (ASCVD) remains a leading cause of death in the United States and developed countries worldwide (1). Although elevated plasma LDL-C continues to be a primary and significant risk factor for ASCVD (2), plasma triglyceride (TG) has also emerged as an independent risk factor (3). In recent decades, the dramatic increase in hypertriglyceridemia associated with diabetes, obesity, and other metabolic disorders threatens to put more patients at risk for ASCVD (4). This has prompted the development of a new generation of therapeutics for cardiovascular disease that can lower plasma TG as well as reduce broader non–HDL-C pools in addition to LDL-C. One potential target that has demonstrated great promise in recent years is angiopoietin-like protein 3 or ANGPTL3.

ANGPTL3 is a member of the angiopoietin-like protein family, a group of secreted proteins that share structural similarity to the angiopoietins, key factors that regulate angiogenesis (5). ANGPTL3 is a 70 kDa protein that is principally secreted from the liver. Owing to its function as an inhibitor of LpL and endothelial lipase (EL), ANGPTL3 is a potent modulator of plasma lipids and can exert effects across all lipoprotein classes (6). A spontaneous loss-of-function (LOF) mutation in an unknown gene, later identified as *Angptl3*, was initially described in hypertriglyceridemic and hyperglycemic obese KK mice (7). This mutation resulted in reductions in plasma TG and elevations in post-heparin LpL activity. Additional studies performed in the KK mice with the *Angptl3* LOF mutation found ANGPTL3 protein deficiency was also associated with lower levels of HDL-C and HDL-phospholipid (8). This effect on HDL was attributed to ANGPTL3 being an inhibitor of EL as well as LpL activity. These observations from mice were extended to humans where LOF mutations in *Angptl3* can result in a condition called familial combined hypobetalipoproteinemia (FHBL2). FHBL2 is a condition characterized by low levels of all the major lipoprotein classes (VLDL, LDL, and HDL) (9). Recently, ANGPTL3 gained even more interest as a therapeutic target when carriers of *Angptl3* LOF mutations were demonstrated to have a significant protection from ASCVD (10, 11). Consistent with this, studies in animal models using genetic, antisense, and antibody-mediated inhibition of ANGPTL3 demonstrated significant reduction in atherosclerotic plaque formation (11, 12, 13).

HDL-C has been shown to have an inverse relationship with ASCVD risk (14). The primary mechanism hypothesized is by facilitating reverse cholesterol transport (RCT), a process by which effluxed cholesterol from peripheral tissues is delivered to the liver for excretion as bile or biliary cholesterol (14). As mentioned previously, preclinical studies have established the reduction in HDL-C associated with genetic or pharmacological inhibition of ANGPTL3 is due to activation of EL (6, 8). However, the consequences of ANGPTL3 inhibition on HDL-mediated RCT has not been fully evaluated. Therefore, to determine the effect of ANGPTL3 inhibition on HDL-C and RCT, we performed a series of dose-response and RCT experiments with an antisense oligonucleotide (ASO) targeting *Angptl3* in mouse models with dramatically different lipoprotein profiles, ranging from mildly to severely hyperlipidemic. Our data revealed that inhibition of ANGPTL3 reduced HDL-C in models where HDL was the predominate lipoprotein class; however, in a mouse model with elevated plasma apoB-containing lipoproteins, reduction of ANGPTL3 had no observed effect on HDL-C levels. Interestingly, in all models tested, inhibition of ANGPTL3 enhanced macrophage-to-feces RCT. Additional studies evaluating HDL clearance confirmed that inhibition of ANGPTL3 improved RCT. These results suggest that inhibition of ANGPTL3 can not only reduce levels of proatherogenic lipoproteins but also enhance a potentially protective function of HDL, irrespective of the effect on HDL-C.

## Materials and methods

### Antisense oligonucleotides

Uniform chimeric 20-mer phosphorothioate oligonucleotides containing 2′-*O*-methoxyethyl groups at positions 1 to 5 and 15 to 20 targeted to murine *Angptl3*, and a control ASO was synthesized and purified on an automated DNA synthesizer using phosphoramidite chemistry as previously described (15). The ASO sequences were as follows: *Angptl3* ASO-ION 595352 (5′-TTTCTTTTATCTGCATGTGC-3′) and control ASO-ION 141923 (5′- AGCATAGTTAACGAGCTCCC-3′), with underlined sequences indicating 2′-*O*-methoxyethyl–modified bases.

### Mice and diets

The wild-type and LDLr−/− mice on a C57BL/6 background were purchased from Jackson Laboratory (Bar Harbor, ME). The human cholesteryl ester transfer protein (*CETP*) transgenic (tg) mice used in these studies were a gift from the laboratory of Linda Curtiss, and the generation of these mice was previously described in detail (16). The *CETP* tg, LDLr−/− mice were generated by breeding the *CETP* tg mice with LDLr−/− mice. The resulting heterozygous animals were backcrossed to generate homozygous *CETP* tg, LDLr−/− mice. Mice were housed in groups of 3 to 5 on a 12-h light-dark cycle for the duration of the studies, and all procedures and protocols were approved by an institutional animal care and use committee. For all experiments, mice were switched from chow to a Western diet (WD), Envigo Diet 88137, consisting of 42% of calories as fat and 0.15% cholesterol, a week before baseline plasma samples were drawn by a retro-orbital bleed. Mice in the treatment groups were randomized according to baseline plasma lipids and body weight before the initiation of the experiment.

### Plasma chemistry and lipoprotein analysis

Plasma lipid and transaminase concentrations were analyzed on an Olympus AU400e automated clinical chemistry analyzer (Melville, NY). HDL-C concentration was determined using the manual HDL Cholesterol E Kit from FUJIFILM Wako Diagnostics (Mountain View, CA). Briefly, 75 μl of plasma was diluted 1:1 with phosphotungstate-magnesium salt, and the mixture was incubated at room temperature for 10 min to precipitate apoB-bound lipoproteins. The samples were then spun at 3,000 *g* for 15 min to pellet apoB-bound lipoproteins. The supernatant containing HDL was collected, and the samples were respun and the supernatant reisolated to ensure no contamination from VLDL/LDL. HDL-C was measured by colorimetric assay according to the protocol included in the kit. Plasma ANGPTL3 protein was measured using an ANGPTL3 ELISA kit from R&D Systems (Minneapolis, MN).

### Radiolabeled human HDL3

Human HDL_3_ was labeled with ^3^H-cholesteryl hexadecyl ether (^3^H-CHE) according to previously published techniques (17). Briefly, 1 mCi of ^3^H-CHE in toluene (New England Nuclear) was evaporated under N_2_ and resuspended in 50 μl ethanol. The radioisotope was added dropwise to isolated human HDL purchased from Millipore (Frederick, MD). After the addition of 200 mg of heat-inactivated LPDS, the solution was incubated overnight at 37°C. The HDL_3_ was isolated and concentrated by ultracentrifugation, and the samples were dialyzed in three exchanges of PBS at 4°C for two 4-hour periods and then overnight.

### Comparative pharmacology studies

Eight- to ten-week-old C57BL/6 wild-type, LDLr−/−, and *CETP* tg LDLr−/− mice were administered PBS, control ASO (50 mg/kg/wk), or *Angptl3* ASO at 50, 15, 5, and 1.5 mg/kg per week for 6 weeks by intraperitoneal injection. After the 6-week treatment period, mice were fasted for 4 h and a terminal plasma sample was collected via a heart puncture along with a liver sample for further analysis.

### In vivo reverse cholesterol transport assay

The macrophage-to-feces in vivo RCT assay was performed according to previously described methods (18). Wild-type C57BL/6 and LDLr−/− mice were administered either control or *Angptl3* ASO at 50 mg/kg/wk for 6 weeks. After the ASO treatment phase, mice were intraperitoneally injected with ^3^H-cholesterol-labeled J774A macrophages (approximately 4.79 mil dpm/5.26 mil cells/mouse). In a separate experiment, *CETP* tg, LDLr−/− mice were administered either control ASO or *Angptl3* ASO at 15 mg/kg/wk for 6 weeks. After ASO treatment, mice were intraperitoneally injected with ^3^H-cholesterol-labeled macrophages (approximately 2.52 mil dpm/6.5 mil cells/mouse). For both studies, mice were singly housed in wire-bottom cages for 72 h, plasma samples were taken at 24 and 48 h, and feces were collected over the entire 72 h period. After 72 h, mice were sacrificed, and terminal plasma samples were collected along with liver samples. A 20 μl aliquot of plasma from each time point/mouse was counted for dpm by liquid scintillation counting (LSC). Liver tissues were extracted according to previously described methods (19). The isolated lipid extracts were dried under N_2_ before resuspension in the scintillation cocktail and counted by LSC. Finally, the amount of radiolabeled fecal cholesterol and bile acid was assayed according to previously published methods (18, 20).

### Radiolabeled human HDL3 clearance study

The in vivo radiolabeled HDL clearance study was performed according to previously published methods (21). Wild-type C57BL/6 and LDLr−/− mice were administered either control or *Angptl3* ASO at 50 mg/kg/wk for 6 weeks. After the ASO treatment phase, mice were intravenously injected with ^3^H-CHE-labeled HDL_3_ (approximately 0.45 mil dpm/mouse). Plasma samples were then collected via retro-orbital bleed 5 min, 1, 3, 6, and 24 h after injection. After the 24-h plasma collection, the study was terminated, and livers were perfused with PBS to reduce blood contamination. A 5-μl aliquot of plasma from each time point/mouse was counted for dpm by LSC. Liver tissues were extracted according to previously described methods (19). The isolated lipid extracts were dried under N_2_ before being resuspended in scintillation cocktail and counted by LSC. Plasma dpm was normalized to the 5 min time point.

### qPCR analysis

RNA from liver samples was purified using the Qiagen RNeasy kit (Germantown, MD) and subjected to RT-PCR analysis. The Applied Biosystems (Foster City, CA) StepOnePlus RT-PCR system, which uses real-time fluorescence RT-PCR detection, was used to quantify ASO-mediated reductions in *Angptl3* mRNA. RNA transcripts were normalized to total RNA levels using the control gene cyclophilin A. The sequences of the primer-probe sets used are as follows: mouse *Angptl3*: forward- TCAAGATTTGCTATGTTGGATGATG, reverse- TTATGGACAAAATCTTTAAGTCCATGAC, and probe- AAAATTTTAGCGAATGGCCTCCTGCAGCT and mouse cyclophilin A: forward- TCGCCGCTTGCTGCA, reverse- ATCGGCCGTGATGTCGA, and probe- CCATGGTCAACCCCACCGTGTTC.

### Statistical analysis

All values reported are normally distributed and expressed as the mean ± SEM. To determine statistical significance, one-way ANOVA with Tukey's *post hoc* test was carried out using GraphPad Prism 8™ software with statistical significance set at a two-tailed *P* < 0.05. The fractional catabolic rate (FCR) of radiolabeled HDL from plasma was calculated using previously published methods (22), and results are presented as the mean ± SEM. The FCR results from C57Bl/6 and LDLr−/− mice treated with either control or *Angptl3* ASO was analyzed by paired *t* test using GraphPad Prism 8™ software with statistical significance set at a two-tailed *P* < 0.05.

## Results

### Administration of an *Angptl3* ASO produced dose-responsive reductions in TPC and TG, and variable effects on HDL-C

The effect of ASO-mediated inhibition of *Angptl3* on plasma lipids and lipoproteins was evaluated in an array of mouse models with a wide range of plasma lipids ranging from moderate to severely hyperlipidemic. The WD-fed C57BL/6 wild-type mice had the lowest levels of TPC and TG with HDL as the predominant lipoprotein class. The WD-fed LDLr−/− mice had elevated levels of TPC and TG and high levels of apoB-containing lipoproteins, primarily LDL. The WD-fed *CETP* tg, LDLr−/− mice presented with the highest concentrations of total plasma cholesterol and TG with extremely low to undetectable HDL-C. The lipoprotein profiles of the *CETP* tg, LDLr−/− mice revealed elevations in both VLDL and LDL cholesterol (data not shown). The wide range of plasma lipids and HDL-C across these different models enabled us to determine the effect of ANGPTL3 inhibition on HDL-C and HDL function in context of different lipidemic states.

Dose-response studies were performed with the *Angptl3* ASO in WD-fed C57BL/6 mice, and the results are shown in Table 1. Administration of the *Angptl3* ASO produced dose-dependent reductions in hepatic *Angptl3* mRNA and circulating ANGPTL3 protein. Mice administered the highest dose of 50 mg/kg/wk had the greatest reduction in *Angptl3* mRNA (83%) and protein (68%). Of the different models evaluated, the C57BL/6 mice had the lowest levels of TPC (153 mg/dl) and TG (59 mg/dl) and the highest levels of HDL-C (118 mg/dl) for the PBS group. The greatest reduction in TPC was observed in the 15 mg/kg/wk *Angptl3* ASO group, which was 27% and 35% when compared with the PBS and control ASO groups, respectively. Mice in the 50 and 5 mg/kg/wk *Angptl3* ASO groups also displayed a significant reduction in TPC compared with the control ASO group. Trends for a reduction in plasma TG were observed across mice administered the higher doses of *Angptl3* ASO; however, the reduction in TG (37% vs. PBS group) was only statistically significant at the 50 mg/kg/wk dose group. The reduction in HDL-C was only statistically significant in the 50, 15, and 5 mg/kg/wk *Angptl3* ASO groups when compared with the control ASO group. The greatest reduction in HDL-C was observed in the 15 mg/kg/wk group, which was 29% and 40% compared with the PBS and control ASO groups, respectively.

**Table 1:** Effect of ANGPTL3 inhibition on plasma lipids, *Angptl3* mRNA, and protein in C57BL/6 mice

Treatment Group	TPC (mg/dl)	TG (mg/dl)	HDL-C (mg/dl)	*Angptl3* mRNA (%PBS)	ANGPTL3 Protein (ng/ml)
PBS	154 ± 8	59 ± 5	118 ± 7	100 ± 15	236 ± 13
Control ASO (50 mg/kg)	174 ± 8	56 ± 2	140 ± 7	139 ± 25	355 ± 32^a^
*Angptl3ASO* (50 mg/kg)	126 ± 5^b^	37 ± 2^a^^,^^b^	97 ± 5^b^	17 ± 3^a^^,^^b^	75 ± 4^a^^,^^b^
*Angptl3ASO* (15 mg/kg)	113 ± 6^a^^,^^b^	50 ± 5	84 ± 7^b^	48 ± 5^b^	114 ± 9^a^^,^^b^
*Angptl3ASO* (5 mg/kg)	134 ± 11^b^	52 ± 4	101 ± 10^b^	123 ± 14	247 ± 19^b^
*Angptl3ASO* (1.5 mg/kg)	174 ± 13	61 ± 6	135 ± 13	149 ± 21	311 ± 43

Values represent the mean ± SEM, n = 4–6/group of treatment.

a Denotes a significant difference (*P* < 0.05) compared with PBS.

b Denotes a significant difference (*P* < 0.05) compared with control ASO.

Dose-response studies were also performed with the *Angptl3* ASO in WD-fed LDLr−/− mice, and the results are shown in Table 2. Compared with the wild-type C57BL/6 mice, the LDLr−/− mice had elevated TPC (1,405 mg/dl) and TG levels (442 mg/dl), but lower levels of HDL-C (76 mg/dl) for the PBS group. Consistent with the data from the C57BL/6 experiment, treatment with the *Angptl3* ASO produced dose-responsive reductions in ANGPTL3 mRNA and protein, and the decreases in the 50 and 15 mg/kg groups were statistically significant when compared with the PBS and control ASO groups. The greatest reduction in *Angptl3* mRNA and protein was observed in mice receiving the 50 mg/kg/wk dose, which demonstrated an 89% and 70% decrease when relative to the PBS group. Similar to *Angptl3* mRNA and protein suppression, treatment with the *Angptl3* ASO produced dose-responsive reductions in TPC and TG. These reductions achieved statistical significance in the 50 and 15 mg/kg/wk treatment groups compared with both PBS and control ASO groups. The 50 mg/kg/wk treatment group had the greatest reductions in TPC (68%) and TG (85%) when compared with the PBS group. Interestingly, in contrast to what was observed in the C57BL/6 mice, we did not see changes in HDL-C.

**Table 2:** Effect of ANGPTL3 inhibition on plasma lipids, *Angptl3* mRNA, and protein in LDLr−/− mice

Treatment Group	TPC (mg/dl)	TG (mg/dl)	HDL-C (mg/dl)	*Angptl3* mRNA (%PBS)	ANGPTL3 Protein (ng/ml)
PBS	1,405 ± 145	442 ± 40	76 ± 3	100 ± 14	307 ± 23
Control ASO (50 mg/kg)	1,322 ± 96	454 ± 42	72 ± 4	102 ± 14	385 ± 31
*Angptl3 ASO* (50 mg/kg)	451 ± 26^a^^,^^b^	66 ± 6^a^^,^^b^	72 ± 4	12 ± 3^a^^,^^b^	121 ± 9^a^^,^^b^
*Angptl3ASO* (15 mg/kg)	895 ± 91^a^^,^^b^	178 ± 28^a^^,^^b^	84 ± 4	39 ± 6^a^^,^^b^	199 ± 22^a^^,^^b^
*Angptl3ASO* (5 mg/kg)	995 ± 69	275 ± 54	73 ± 6	62 ± 12	271 ± 13^b^
*Angptl3ASO* (1.5 mg/kg)	1,508 ± 108	572 ± 84	74 ± 2	97 ± 16	338 ± 26

Values represent the mean ± SEM, n = 4–6/group of treatment.

a Denotes a significant difference (*P* < 0.05) compared with PBS.

b Denotes a significant difference (*P* < 0.05) compared with control ASO.

Finally, the plasma lipids and lipoproteins were evaluated in a dose-response study in the WD-fed *CETP* tg, LDLr−/− mice treated with control and *Angptl3* ASO (Table 3). The *CETP* tg, LDLr−/− mice had the highest levels of TPC and TG of all the models tested, with mean values of 2,896 and 782 mg/dl for the PBS group, respectively, and the lowest levels of HDL-C, at 1.8 mg/dl for the PBS group. Administration of the *Angptl3* ASO in these mice resulted in dose-dependent reductions in ANGPTL3 mRNA and protein. The reductions were statically significant in mice receiving the 50 and 15 mg/kg/wk doses of ASO when compared with the PBS and control ASO groups. The greatest reductions were observed at the 50 mg/kg/wk dose, which were 89% and 70% for ANGPTL3 mRNA and protein, respectively. As expected, treatment with the *Angptl3* ASO produced dose-dependent reductions in TPC and TG reaching significance at both the 50 and 15 mg/kg/wk groups relative to the PBS and control ASO animals. The greatest reduction in TPC and TG was observed in the 50 mg/kg/wk treatment group, and these mice displayed 82% and 92% reductions in TPC and TG, respectively, when compared with the PBS group. HDL-C levels were significantly increased to that observed for wild-type C57BL/6 mice receiving the 50 and 15 mg/kg/wk doses of *Angptl3* ASO. The *CETP* tg, LDLr−/− mice receiving either 50 or 15 mg/kg/wk of *Angptl3* ASO had HDL-C levels of 64 mg/dl, a 32- and 9-fold increase in HDL-C when compared with the PBS and control ASO groups, respectively.

**Table 3:** Effect of ANGPTL3 inhibition on plasma lipids, *Angptl3* mRNA, and protein in *CETP* tg, LDLr−/− mice

Treatment Group	TPC (mg/dl)	TG (mg/dl)	HDL-C (mg/dl)	*Angptl3* mRNA (%PBS)	ANGPTL3 Protein (ng/ml)
PBS	2,896 ± 223	782 ± 65	1.8 ± 0.9	100 ± 14	259 ± 20
Control ASO (50 mg/kg)	2,654 ± 189	683 ± 99	7.4 ± 5.5	70 ± 6	263 ± 13
*Angptl3* ASO (50 mg/kg)	522 ± 89^a^^,^^b^	62 ± 3^a^^,^^b^	64 ± 8^a^^,^^b^	11 ± 0.7^a^^,^^b^	79 ± 7^a^^,^^b^
*Angptl3* ASO (15 mg/kg)	783 ± 25^a^^,^^b^	97 ± 8^a^^,^^b^	64 ± 4^a^^,^^b^	25 ± 2^a^^,^^b^	161 ± 9^a^^,^^b^
*Angptl3* ASO (5 mg/kg)	2053 ± 119	413 ± 7	0.5 ± 0.15	64 ± 3	209 ± 4
*Angptl3* ASO (1.5 mg/kg)	2,493 ± 102	548 ± 39	0.05 ± 0.5	102 ± 6	283 ± 6

Values represent the mean ± SEM, n = 3–6/group of treatment.

a Denotes a significant difference (*P* < 0.05) compared with PBS.

b Denotes a significant difference (*P* < 0.05) compared with control ASO.

### RCT was increased in mice treated with the *Angptl3* ASO

**Fig. 1:**
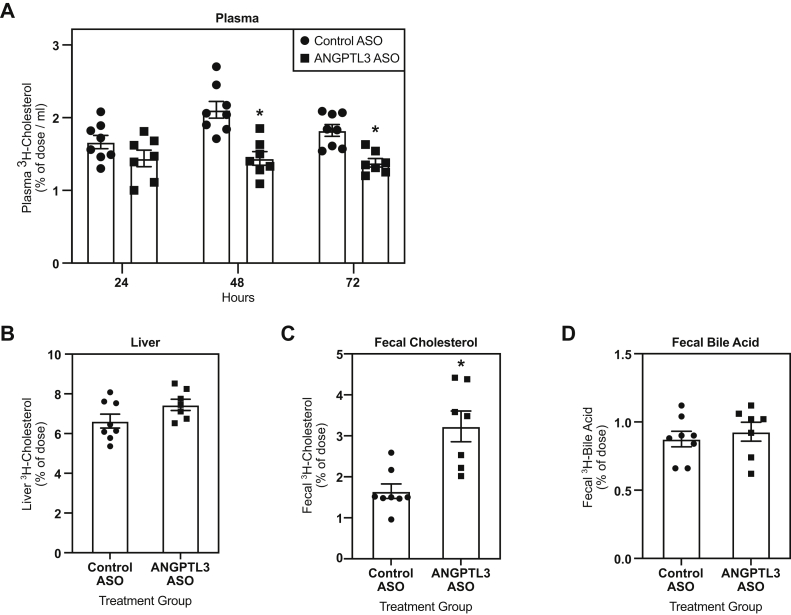
Effect of *Angptl3* ASO on reverse cholesterol transport in WD-fed C57BL/6 mice. A: Clearance of ^3^H-cholesterol from plasma over a 72-h period in mice treated with either control or *Angptl3* ASO for 6 weeks and intraperitoneally injected with radiolabeled macrophages. Amount of ^3^H-cholesterol in (B) the liver, (C) feces, and (D) fecal bile acids (n = 8 for control ASO and n = 7 for *Angptl3* ASO groups). The asterisk denotes a significant difference (*P* < 0.05) between control ASO and *Angptl3* ASO treatment groups. ANGPTL3, angiopoietin-like protein 3; ASO, antisense oligonucleotide; WD, Western diet.

**Fig. 2:**
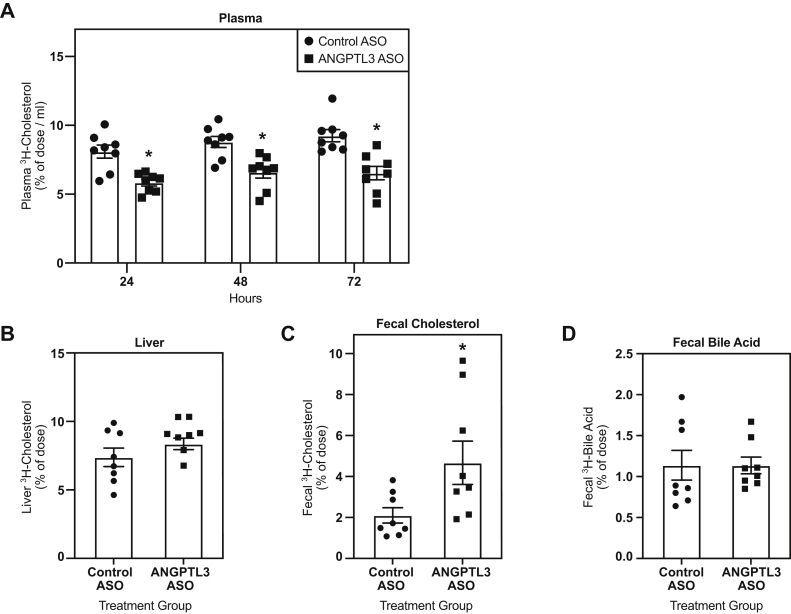
Effect of *Angptl3* ASO on reverse cholesterol transport in WD-fed LDLr−/− mice. A: Clearance of ^3^H-cholesterol from plasma over a 72-h period in mice treated with either control or *Angptl3* ASO for 6 weeks and intraperitoneally injected with radiolabeled macrophages. The amount of ^3^H-cholesterol in (B) the liver, (C) feces, and (D) fecal bile acids (n = 8 for control ASO and *Angptl3* ASO groups). The asterisk denotes a significant difference (*P* < 0.05) between control ASO and *Angptl3* ASO treatment groups. ANGPTL3, angiopoietin-like protein 3; ASO, antisense oligonucleotide; WD, Western diet.

**Fig. 3:**
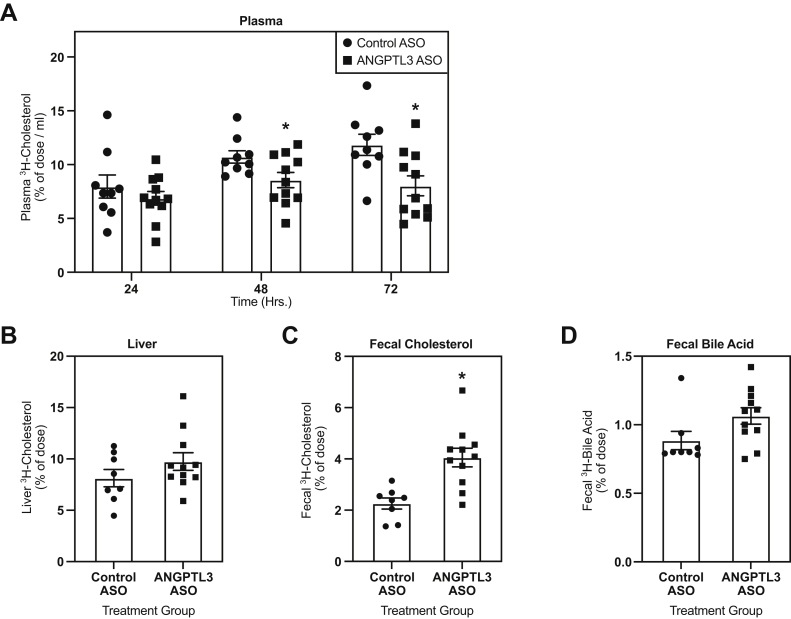
Effect of *Angptl3* ASO on reverse cholesterol transport in WD-fed *CETP* tg, LDLr−/− mice. A: Clearance of ^3^H-cholesterol from plasma over a 72-h period in mice treated with either control or *Angptl3* ASO for 6 weeks and intraperitoneally injected with radiolabeled macrophages. The amount of ^3^H-cholesterol in (B) the liver, (C) feces, and (D) fecal bile acids (n = 9 for control ASO and n = 11 *Angptl3* ASO groups). The asterisk denotes a significant difference (*P* < 0.05) between control ASO and *Angptl3* ASO treatment groups. ANGPTL3, angiopoietin-like protein 3; ASO, antisense oligonucleotide; CETP, cholesteryl ester transfer protein; WD, Western diet.

Next, the effect of ASO-mediated inhibition of *Angptl3* on in vivo RCT was evaluated in WD-fed C57BL/6, LDLr−/−, and *CETP* tg, LDLr−/− mice treated with either control or *Angptl3* ASO for 6 weeks. These studies used the maximally efficacious dose of 50 mg/kg/wk identified from the dose-response studies. C57BL/6 mice treated with the *Angptl3* ASO displayed a significant reduction in the amount of radiolabeled cholesterol in plasma 48 (32%) and 72 h (25%) after injection of radiolabeled macrophages versus control ASO (Fig. 1A). In addition, C57BL/6 mice treated with the *Angptl3* ASO had a 96% increase in radiolabeled fecal cholesterol compared with the control ASO group (Fig. 1C). Similarly, LDLr−/− mice treated with the *Angptl3* ASO had a reduction in the accumulation of radiolabeled cholesterol in plasma when compared with the control ASO group (Fig. 2A). However, the reduction was significant at all time points measured, and the decrease in radiolabeled plasma cholesterol was 29%, 25%, and 28% at 24, 48, and 72 h after the injection of radiolabeled macrophages. LDLr−/− mice administered the *Angptl3* ASO also had an increase in radiolabeled fecal cholesterol (122%) when compared with the control ASO group (Fig. 2C). Finally, when RCT was assayed in the *CETP* tg, LDLr−/− mice, these mice also displayed reductions in amount of radiolabeled cholesterol in plasma (Fig. 3A). This reduction achieved statistical significance 48 and 72 h after the injection of the radiolabeled macrophages, which was 20% and 32%, respectively. Similar to what was observed in the other models tested, the *CETP* tg, LDLr−/− mice administered the *Angptl3* ASO displayed a significant increase in fecal cholesterol (79%) relative to the control group (Fig. 3C). In all models evaluated, no significant differences in hepatic radiolabeled cholesterol and fecal bile acid were observed (Fig. 1, Fig. 2, Fig. 3). In summary, in all models evaluated, mice administered the *Angptl3* ASO displayed a decrease in the accumulation of radiolabeled cholesterol in plasma and an increase in the appearance of radiolabeled cholesterol in the feces. These results suggest that inhibition of ANGPTL3 can enhance RCT in mice.

### HDL clearance was enhanced in LDLr−/− mice administered the *Angptl3* ASO

**Fig. 4:**
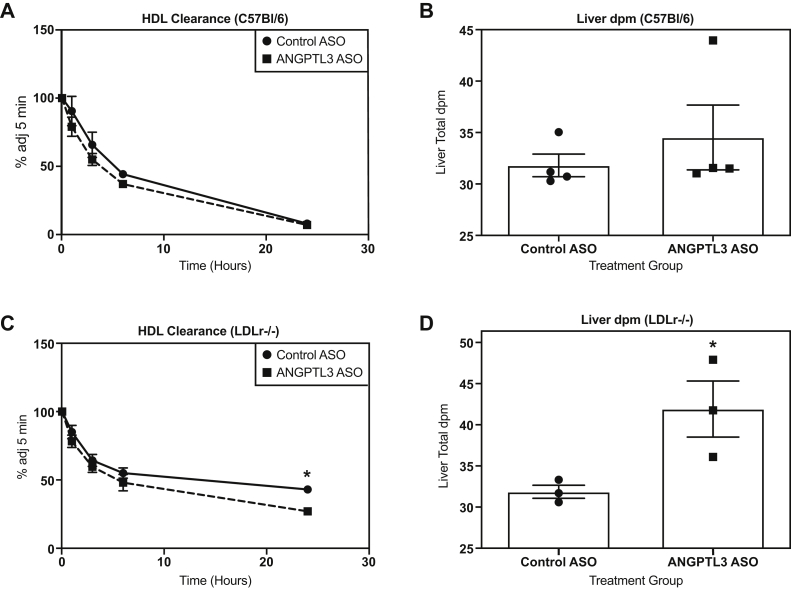
Effect of *Angptl3* ASO on radiolabeled HDL clearance. A: Clearance of HDL-^3^H-CHE from plasma over 24 h in C57Bl/6 mice administered either control or *Angptl3* ASO for 6 weeks before intravenous injection of labeled HDL (n = 4 for control ASO and *Angptl3* ASO groups). B: Amount of ^3^H-CHE in the liver of C57Bl/6 mice treated with either control or *Angptl3* ASO. C: Clearance of HDL-^3^H-CHE from plasma over 24 h in LDLr−/− mice administered either control or *Angptl3* ASO for 6 weeks before intravenous injection of labeled HDL (n = 3 for control ASO and *Angptl3* ASO groups). D: Amount of ^3^H-CHE in the liver of LDLr−/− mice treated with either control or *Angptl3* ASO. The asterisk denotes a significant difference (*P* < 0.05) between control ASO and *Angptl3* ASO treatment groups. ANGPTL3, angiopoietin-like protein 3; ASO, antisense oligonucleotide; ^3^H-CHE, ^3^H-cholesteryl hexadecyl ether.

To gain further insight into the effect of ANGPTL3 inhibition on RCT, HDL clearance assays were performed in WD-fed C57BL/6 and LDLr−/− mice treated with either control or *Angptl3* ASO. Mice were injected with human HDL_3_ that was radiolabeled with ^3^H-CHE. ^3^H-CHE is a modified lipid marker that cannot be exchanged with other lipids nor can it be metabolized, so it will accumulate in tissues such as the liver. In the WD-fed C57BL/6 mice, no significant effect on the plasma FCR of radiolabeled HDL_3_ could be detected between the control and *Angptl3* ASO–treated mice at 0.118 ± 0.007 h^-1^ and 0.138 ± 0.01 h^-1^, respectively (Fig. 4A). Correspondingly, no effect was observed on hepatic accumulation of ^3^H-CHE when compared with the control ASO group (Fig. 4B). This lack of an effect on HDL clearance in the C57BL/6 mice was similar to what was observed in the RCT assay at the 24-h time plasma point, indicating a delay in the induction of RCT. However, in the LDLr−/− mice, the *Angptl3* ASO treatment group did display a significant increase in the plasma FCR of radiolabeled HDL_3_ at 0.0545 ± 0.007 h^-1^ when compared with the control ASO group where the FCR was 0.0232 ± 0.001 h^-1^ (Fig. 4C). The increased clearance of labeled HDL from plasma in the ANGPTL3 group was associated with significantly greater accumulation of ^3^H-CHE (31.5%) in the liver when compared with the control ASO group (Fig. 4D). These results support and extend observations from the RCT assays, suggesting that inhibition of ANGPTL3 can enhance HDL-mediated RCT.

## Discussion

In recent years, ANGPTL3 has emerged as a promising target for ASCVD based on observations from preclinical and clinical studies demonstrating that inhibition of ANGPTL3 can lower proatherogenic lipids and in animal models reduce atherosclerosis. Individuals with *Angptl3* LOF mutations have a lipid phenotype characterized as FHBL2, a condition where all major classes of lipoproteins are reduced (23). Given the lifelong reductions in proatherogenic LDL-C commonly found in subjects with FHBL2, it is highly unlikely that lower levels of HDL-C would significantly contribute to ASCVD progression in this patient population. This concept is supported by the protection from atherosclerosis observed in subjects with FHBL2 (10, 11). However, because ANGPTL3 inhibitors are being developed to treat preexisting hyperlipidemia in broad populations at risk for ASCVD, it is of interest to determine the effect of ANGPTL3 inhibition on not only HDL-C but also HDL function, particularly RCT. To our knowledge, the effect of ANGPTL3 inhibition on in vivo RCT has not been evaluated in experimental models. To determine the effect of ANGPTL3 inhibition on HDL-C and RCT, we performed a series of dose-response and RCT studies with an *Angptl3* ASO in mouse models with different levels of plasma lipids, ranging from moderate to severely hyperlipidemic. *Angptl3* ASO–mediated reductions in HDL-C were limited to the WD-fed C57BL/6 mice, where the majority of plasma cholesterol was associated with HDL. Surprisingly, regardless of the effect on HDL-C, treatment with the *Angptl3* ASO enhanced RCT in all models tested. The observations from the RCT assays were consistent in HDL clearance studies, where WD-fed LDLr−/− mice treated with the *Angptl3* ASO displayed increased plasma clearance and hepatic uptake of labeled HDL. The results from our studies suggest that inhibition of ANGPTL3 can not only reduce levels of proatherogenic lipids but also potentially enhance RCT.

The lower levels of HDL-C commonly observed in subjects with FHBL2 is predicted to result from the derepression of EL phospholipase activity when levels of functional ANGPTL3 are reduced or absent (8, 24). The results from our studies suggest that the effect of genetic or pharmacological inhibition of ANGPTL3 on HDL-C is context dependent, in that levels of non-HDL-C and TG can influence HDL-C. As previously reported in models with moderate levels of plasma cholesterol, reduction of ANGPTL3 mRNA and protein in the WD-fed C57BL/6 mice resulted in significant reductions in HDL-C (8, 24, 25). However, this was not the case in the hyperlipidemic LDLr−/− mice. This discrepancy in HDL-C between these two mouse models raises two possibilities that will require additional study. In the WD-fed C57BL/6 mice, the majority of plasma cholesterol was associated with HDL, while the ratio of non-HDL-C to HDL-C was much higher in the LDLr−/− mice. Investigators have shown that while EL prefers HDL, the enzyme can interact with apoB-containing lipoproteins as well (26, 27). Potentially, the lack of a significant effect on HDL-C observed in the LDLr−/− mice is due to the elevated levels of apoB lipoproteins outcompeting HDL particles for access to EL. Alternatively, the catabolism of TG-rich lipoproteins is enhanced when ANGPTL3 is inhibited or reduced because of activation of LpL (6, 28). Because surface phospholipid released from TG-rich lipoproteins undergoing LpL-mediated lipolysis can serve as a substrate for the formation of nascent pre-β HDL particles (29), perhaps an increase in de novo synthesis of HDL particles can counteract EL-mediated reductions in HDL-C. The net effect resulting in similar levels of HDL-C was observed across the different treatment groups of LDLr−/− mice.

Interestingly, the dose-response study in the WD-fed *CETP* tg, LDLr−/− mice indicates there are also conditions where reductions in ANGPTL3 can increase HDL-C. The PBS-treated and control ASO–treated *CETP* tg, LDLr−/− mice had extremely low levels of HDL-C, with concentrations ranging from 2 to 8 mg/dl, whereas *CETP* tg, LDLr−/− mice administered the 50 and 15 mg/kg doses of *Angptl3* ASO had normalized levels of HDL-C at 64 mg/dl. The increase in HDL-C in *CETP* tg, LDLr−/− mice treated with *Angptl3* ASO demonstrates that in the setting of neutral lipid transfer protein, CETP, and hypertriglyceridemia, the *Angptl3* ASO–mediated reductions in TG can limit the amount of VLDL-TG available for exchange for HDL-cholesteryl ester. The net effect resulted in increases in HDL-C. Similar observations have been made in hyperlipidemic models expressing CETP when administered TG-lowering agents (30, 31, 32). This effect on HDL-C was lost in *CETP* tg, LDLr−/− mice treated with lower levels of the *Angptl3* ASO, suggesting in this model, there is a threshold of TG reduction that must be crossed to observe an increase in HDL-C.

A consistent and intriguing observation from our studies was, regardless of the degree of hyperlipidemia or effects on steady state measurements of plasma HDL, mice administered the *Angptl3* ASO displayed a reduction in the plasma accumulation and an increase in the fecal excretion of radiolabeled cholesterol, suggesting an increase in RCT. Along with anti-inflammatory and antioxidant capabilities, the ability to facilitate RCT is commonly believed to be a key protective function of HDL (33, 34). Presumably, the positive effect on RCT is due to enhanced EL activity when ANGPTL3 protein is reduced (8). The relationships between EL, HDL, and RCT have been extensively studied (35); however, the absolute effect of EL activation across all phases of RCT is still being examined. Investigators have shown increases in EL protein and enzymatic activity can enhance the FCR of HDL and promote cholesterol uptake in the liver and kidney (36). This result is similar to what we observed when LDLr−/− mice treated with *Angptl3* ASO were injected with HDL loaded with radiolabeled cholesteryl ether. However, in C57BL/6 mice treated with *Angptl3* ASO, we did not observe improvements in the plasma clearance and hepatic uptake of labeled HDL after 24 h. Similarly, we also did not detect a significant effect in plasma at the 24-h time point when we performed the macrophage-to-feces RCT assay in C57Bl/6 mice. These results indicate that there is a delay in HDL catabolism in C57Bl/6 mice treated with *Angptl3* ASO; however, why this phenomenon occurs is unclear. Finally, our studies showed an increase in fecal excretion of biliary cholesterol, a result that suggests the final step in RCT is enhanced when ANGPTL3 is reduced. This result was unexpected as previous experiments in mice overexpressing EL did not show an effect on bile or biliary cholesterol excretion (37). Our initial observations will require additional study; however, investigators have shown hepatic lipid secretion is reduced when ANGPTL3 is inhibited (13, 38). Perhaps, in the context of reduced ANGPTL3 protein, a decrease in hepatic lipid secretion could result in an increase in fecal cholesterol excretion to prevent the accumulation of excess cholesterol in the liver.

While we did observe consistent increases in macrophage to feces RCT in all models tested, there are some caveats with the RCT experiments that must be noted that limit translatability of these results to ANGPTL3 inhibitors currently undergoing clinical evaluation. The use of exogenous, cholesterol-loaded macrophages in the RCT assay does not take into account the bidirectional movement of cholesterol in and out of macrophages, thereby potentially minimizing or bypassing the effect of ANGPTL3 inhibition on the first step of RCT, macrophage transfer of cholesterol to HDL. This critique is supported by studies where cholesterol efflux was evaluated in apoB-depleted serum from subjects with FHBL2. Investigators found apoB-depleted serum from homozygous carriers of *Angptl3* mutations had significantly reduced cholesterol efflux capacity, in particular, ABCA1-mediated cholesterol efflux (39, 40). Granted, ANGPTL3 inhibitors will never fully recapitulate the complete loss of ANGPTL3 protein observed in homozygous FHBL2 subjects, the pharmacological effect would most likely be more comparable to the heterozygous condition. Heterozygous carriers of *Angptl3* mutations typically display a more intermediate reduction of cholesterol efflux capacity. Considering the results from the cholesterol efflux studies in FHBL2 subjects, the improvement in RCT observed in mouse models with the *Angptl3* ASO is probably due to downstream effects on RCT, for example, increasing hepatic uptake and fecal elimination of the labeled cholesterol.

The results from our studies raise the exciting possibility that in addition to robust reductions in total plasma cholesterol and TG, ANGPTL3 inhibition can also enhance HDL-mediated RCT, indicating ANGPTL3 suppression can exert antiatherogenic effects across all lipoprotein classes. This positive effect on RCT was consistent across all models tested, although the effect on HDL-C was variable. In general, clinical evaluations of ANGPTL3 therapeutics in normal and hyperlipidemic patients have replicated what have been observed in subjects with FHBL2, that inhibition of ANGPTL3 typically results in a reduction in HDL-C (13, 41, 42). This lowering of HDL-C is generally considered to be an acceptable liability in the context of the significant reductions of proatherogenic lipids also observed when ANGPTL3 is inhibited. HDL is generally considered to confer protection from atherosclerosis. This concept has developed over time from observations from numerous epidemiological studies demonstrating HDL-C level is inversely related to ASCVD risks (43). This theory has been supported by decades of research demonstrating that the cholesterol efflux and anti-inflammatory, antioxidant, and antithrombotic functions of HDL can all contribute to the lipoprotein's antiatherogenic potential (33). The failure of HDL-targeted therapeutics in the clinic has called into question the relative effect of HDL in ASCVD (44). Lessons from the clinic has highlighted the overall challenges with developing HDL-targeted therapeutics. Unlike LDL, where the overall goal of a therapeutic intervention is to decrease this atherogenic lipoprotein as much as possible, HDL are highly dynamic particles and shifts in HDL-C alone likely do not provide enough information to guide drug discovery. The results from our studies assessing the effect of an *Angptl3* ASO on RCT suggest this could be an example where HDL-C does not reflect improvements in HDL function, likely because of the widespread effects ANGPTL3 inhibition has on overall lipoprotein and liver lipid metabolism (6). If our observation that ASO-mediated *Angptl3* inhibition can improve HDL RCT is extended to the clinic, it could create an opportunity to develop sensitive and high-throughput assays to detect changes in HDL-mediated RCT and identify novel approaches for creating HDL-targeted therapeutics.

## Data availability

All data reported in this study are located within the article.

## Conflict of interest

All authors are employees of Ionis Pharmaceuticals.

## References

[1] Roth G.A. (2018). Global, regional, and national age-sex-specific mortality for 282 causes of death in 195 countries and territories, 1980-2017: a systematic analysis for the Global Burden of Disease Study 2017. Lancet.

[2] Ference B.A., Ginsberg H.N., Graham I., Ray K.K., Packard C.J., Bruckert E., Hegele R.A., Krauss R.M., Raal F.J., Schunkert H., Watts G.F., Borén J., Fazio S., Horton J.D., Masana L. (2017). Low-density lipoproteins cause atherosclerotic cardiovascular disease. 1. Evidence from genetic, epidemiologic, and clinical studies. A consensus statement from the European Atherosclerosis Society Consensus Panel. Eur. Heart J..

[3] Sandesara P.B., Virani S.S., Fazio S., Shapiro M.D. (2019). The forgotten lipids: triglycerides, remnant cholesterol, and atherosclerotic cardiovascular disease risk. Endocr. Rev..

[4] Ganda O.P., Bhatt D.L., Mason R.P., Miller M., Boden W.E. (2018). Unmet need for adjunctive dyslipidemia therapy in hypertriglyceridemia management. J. Am. Coll. Cardiol..

[5] Biterova E., Esmaeeli M., Alanen H.I., Saaranen M., Ruddock L.W. (2018). Structures of Angptl3 and Angptl4, modulators of triglyceride levels and coronary artery disease. Sci. Rep..

[6] Tikka A., Jauhiainen M. (2016). The role of ANGPTL3 in controlling lipoprotein metabolism. Endocrine.

[7] Koishi R., Ando Y., Ono M., Shimamura M., Yasumo H., Fujiwara T., Horikoshi H., Furukawa H. (2002). Angptl3 regulates lipid metabolism in mice. Nat. Genet..

[8] Shimamura M., Matsuda M., Yasumo H., Okazaki M., Fujimoto K., Kono K., Shimizugawa T., Ando Y., Koishi R., Kohama T., Sakai N., Kotani K., Komuro R., Ishida T., Hirata K. (2007). Angiopoietin-like protein3 regulates plasma HDL cholesterol through suppression of endothelial lipase. Arterioscler. Thromb. Vasc. Biol..

[9] Minicocci I., Tikka A., Poggiogalle E., Metso J., Montali A., Ceci F., Labbadia G., Fontana M., Di Costanzo A., Maranghi M., Rosano A., Ehnholm C., Donini L.M., Jauhiainen M., Arca M. (2016). Effects of angiopoietin-like protein 3 deficiency on postprandial lipid and lipoprotein metabolism. J. Lipid Res..

[10] Stitziel N.O., Khera A.V., Wang X., Bierhals A.J., Vourakis A.C., Sperry A.E., Natarajan P., Klarin D., Emdin C.A., Zekavat S.M., Nomura A., Erdmann J., Schunkert H., Samani N.J., Kraus W.E. (2017). ANGPTL3 deficiency and protection against coronary artery disease. J. Am. Coll. Cardiol..

[11] Dewey F.E., Gusarova V., Dunbar R.L., O'Dushlaine C., Schurmann C., Gottesman O., McCarthy S., Van Hout C.V., Bruse S., Dansky H.M., Leader J.B., Murray M.F., Ritchie M.D., Kirchner H.L., Habegger L. (2017). Genetic and pharmacologic inactivation of ANGPTL3 and cardiovascular disease. N. Engl. J. Med..

[12] Ando Y., Shimizugawa T., Takeshita S., Ono M., Shimamura M., Koishi R., Furukawa H. (2003). A decreased expression of angiopoietin-like 3 is protective against atherosclerosis in apoE-deficient mice. J. Lipid Res..

[13] Graham M.J., Lee R.G., Brandt T.A., Tai L.J., Fu W., Peralta R., Yu R., Hurh E., Paz E., McEvoy B.W., Baker B.F., Pham N.C., Digenio A., Hughes S.G., Geary R.S. (2017). Cardiovascular and metabolic effects of ANGPTL3 antisense oligonucleotides. N. Engl. J. Med..

[14] Rosenson R.S., Brewer H.B., Barter P.J., Björkegren J.L.M., Chapman M.J., Gaudet D., Kim D.S., Niesor E., Rye K.A., Sacks F.M., Tardif J.C., Hegele R.A. (2018). HDL and atherosclerotic cardiovascular disease: genetic insights into complex biology. Nat. Rev. Cardiol..

[15] Ravikumar V.T., Andrade M., Carty R.L., Dan A., Barone S. (2006). Development of siRNA for therapeutics: efficient synthesis of phosphorothioate RNA utilizing phenylacetyl disulfide (PADS). Bioorg. Med. Chem. Lett..

[16] Agellon L.B., Walsh A., Hayek T., Moulin P., Jiang X.C., Shelanski S.A., Breslow J.L., Tall A.R. (1991). Reduced high density lipoprotein cholesterol in human cholesteryl ester transfer protein transgenic mice. J. Biol. Chem..

[17] Lagor W.R., Brown R.J., Toh S.A., Millar J.S., Fuki I.V., de la Llera-Moya M., Yuen T., Rothblat G., Billheimer J.T., Rader D.J. (2009). Overexpression of apolipoprotein F reduces HDL cholesterol levels in vivo. Arterioscler. Thromb. Vasc. Biol..

[18] Zhang Y., Zanotti I., Reilly M.P., Glick J.M., Rothblat G.H., Rader D.J. (2003). Overexpression of apolipoprotein A-I promotes reverse transport of cholesterol from macrophages to feces in vivo. Circulation.

[19] Bligh E.G., Dyer W.J. (1959). A rapid method of total lipid extraction and purification. Can. J. Biochem. Physiol..

[20] Temel R.E., Sawyer J.K., Yu L., Lord C., Degirolamo C., McDaniel A., Marshall S., Wang N., Shah R., Rudel L.L., Brown J.M. (2010). Biliary sterol secretion is not required for macrophage reverse cholesterol transport. Cell Metab..

[21] Spady D.K., Woollett L.A., Meidell R.S., Hobbs H.H. (1998). Kinetic characteristics and regulation of HDL cholesteryl ester and apolipoprotein transport in the apoA-I-/- mouse. J. Lipid Res..

[22] Lee J.Y., Lanningham-Foster L., Boudyguina E.Y., Smith T.L., Young E.R., Colvin P.L., Thomas M.J., Parks J.S. (2004). Prebeta high density lipoprotein has two metabolic fates in human apolipoprotein A-I transgenic mice. J. Lipid Res..

[23] Welty F.K. (2014). Hypobetalipoproteinemia and abetalipoproteinemia. Curr. Opin. Lipidol..

[24] Gusarova V., Alexa C.A., Wang Y., Rafique A., Kim J.H., Buckler D., Mintah I.J., Shihanian L.M., Cohen J.C., Hobbs H.H., Xin Y., Valenzuela D.M., Murphy A.J., Yancopoulos G.D., Gromada J. (2015). ANGPTL3 blockade with a human monoclonal antibody reduces plasma lipids in dyslipidemic mice and monkeys. J. Lipid Res..

[25] Xu Y.X., Redon V., Yu H., Querbes W., Pirruccello J., Liebow A., Deik A., Trindade K., Wang X., Musunuru K., Clish C.B., Cowan C., Fizgerald K., Rader D., Kathiresan S. (2018). Role of angiopoietin-like 3 (ANGPTL3) in regulating plasma level of low-density lipoprotein cholesterol. Atherosclerosis.

[26] Broedl U.C., Maugeais C., Millar J.S., Jin W., Moore R.E., Fuki I.V., Marchadier D., Glick J.M., Rader D.J. (2004). Endothelial lipase promotes the catabolism of ApoB-containing lipoproteins. Circ. Res..

[27] McCoy M.G., Sun G.S., Marchadier D., Maugeais C., Glick J.M., Rader D.J. (2002). Characterization of the lipolytic activity of endothelial lipase. J. Lipid Res..

[28] Shimizugawa T., Ono M., Shimamura M., Yoshida K., Ando Y., Koishi R., Ueda K., Inaba T., Minekura H., Kohama T., Furukawa H. (2002). ANGPTL3 decreases very low density lipoprotein triglyceride clearance by inhibition of lipoprotein lipase. J. Biol. Chem..

[29] Lewis G.F., Rader D.J. (2005). New insights into the regulation of HDL metabolism and reverse cholesterol transport. Circ. Res..

[30] van der Hoorn J.W., Jukema J.W., Havekes L.M., Lundholm E., Camejo G., Rensen P.C., Princen H.M. (2009). The dual PPARalpha/gamma agonist tesaglitazar blocks progression of pre-existing atherosclerosis in APOE∗3Leiden.CETP transgenic mice. Br. J. Pharmacol..

[31] Hansen M.K., McVey M.J., White R.F., Legos J.J., Brusq J.M., Grillot D.A., Issandou M., Barone F.C. (2010). Selective CETP inhibition and PPARalpha agonism increase HDL cholesterol and reduce LDL cholesterol in human ApoB100/human CETP transgenic mice. J. Cardiovasc. Pharmacol. Ther..

[32] Kühnast S., Louwe M.C., Heemskerk M.M., Pieterman E.J., van Klinken J.B., van den Berg S.A., Smit J.W., Havekes L.M., Rensen P.C., van der Hoorn J.W., Princen H.M., Jukema J.W. (2013). Niacin Reduces Atherosclerosis Development in APOE∗3Leiden.CETP Mice Mainly by Reducing NonHDL-Cholesterol. PLoS One.

[33] Rosenson R.S., Brewer H.B., Ansell B.J., Barter P., Chapman M.J., Heinecke J.W., Kontush A., Tall A.R., Webb N.R. (2016). Dysfunctional HDL and atherosclerotic cardiovascular disease. Nat. Rev. Cardiol..

[34] Rosenson R.S., Brewer H.B., Davidson W.S., Fayad Z.A., Fuster V., Goldstein J., Hellerstein M., Jiang X.C., Phillips M.C., Rader D.J., Remaley A.T., Rothblat G.H., Tall A.R., Yvan-Charvet L. (2012). Cholesterol efflux and atheroprotection: advancing the concept of reverse cholesterol transport. Circulation.

[35] Yasuda T., Ishida T., Rader D.J. (2010). Update on the role of endothelial lipase in high-density lipoprotein metabolism, reverse cholesterol transport, and atherosclerosis. Circ. J..

[36] Maugeais C., Tietge U.J., Broedl U.C., Marchadier D., Cain W., McCoy M.G., Lund-Katz S., Glick J.M., Rader D.J. (2003). Dose-dependent acceleration of high-density lipoprotein catabolism by endothelial lipase. Circulation.

[37] Wiersma H., Gatti A., Nijstad N., Kuipers F., Tietge U.J. (2009). Hepatic SR-BI, not endothelial lipase, expression determines biliary cholesterol secretion in mice. J. Lipid Res..

[38] Wang Y., Gusarova V., Banfi S., Gromada J., Cohen J.C., Hobbs H.H. (2015). Inactivation of ANGPTL3 reduces hepatic VLDL-triglyceride secretion. J. Lipid Res..

[39] Pisciotta L., Favari E., Magnolo L., Simonelli S., Adorni M.P., Sallo R., Fancello T., Zavaroni I., Ardigò D., Bernini F., Calabresi L., Franceschini G., Tarugi P., Calandra S., Bertolini S. (2012). Characterization of three kindreds with familial combined hypolipidemia caused by loss-of-function mutations of ANGPTL3. Circ. Cardiovasc. Genet..

[40] Minicocci I., Cantisani V., Poggiogalle E., Favari E., Zimetti F., Montali A., Labbadia G., Pigna G., Pannozzo F., Zannella A., Ceci F., Ciociola E., Santini S., Maranghi M., Vestri A. (2013). Functional and morphological vascular changes in subjects with familial combined hypolipidemia: an exploratory analysis. Int. J. Cardiol..

[41] Gaudet D., Karwatowska-Prokopczuk E., Baum S.J., Hurh E., Kingsbury J., Bartlett V.J., Figueroa A.L., Piscitelli P., Singleton W., Witztum J.L., Geary R.S., Tsimikas S., St L O’Dea L. (2020). Vupanorsen, an N-acetyl galactosamine-conjugated antisense drug to ANGPTL3 mRNA, lowers triglycerides and atherogenic lipoproteins in patients with diabetes, hepatic steatosis, and hypertriglyceridaemia. Eur. Heart J..

[42] Raal F.J., Rosenson R.S., Reeskamp L.F., Hovingh G.K., Kastelein J.J.P., Rubba P., Ali S., Banerjee P., Chan K.C., Gipe D.A., Khilla N., Pordy R., Weinreich D.M., Yancopoulos G.D., Zhang Y. (2020). Evinacumab for Homozygous Familial Hypercholesterolemia. N. Engl. J. Med..

[43] Rader D.J., Hovingh G.K. (2014). HDL and cardiovascular disease. Lancet.

[44] Tall A.R., Rader D.J. (2018). Trials and Tribulations of CETP Inhibitors. Circ. Res..

